# A quantitative approach to intravenous fluid therapy in the syndrome of inappropriate antidiuretic hormone secretion

**DOI:** 10.1007/s10157-019-01741-6

**Published:** 2019-05-02

**Authors:** Philip J. G. M. Voets, Nils P. J. Vogtländer

**Affiliations:** Department of Nephrology, Gelre Hospital, Albert Schweitzerlaan 31, 7334 DZ Apeldoorn, The Netherlands

**Keywords:** Intravenous fluid therapy, Serum sodium concentration, Antidiuretic hormone, SIADH, Formula, Equation

## Abstract

**Background:**

A wide range of interesting mathematical models has been derived to predict the effect of intravenous fluid therapy on the serum sodium concentration (most notably the Adrogué–Madias equation), but unfortunately, these models cannot be applied to patients with disorders characterized by aberrant antidiuretic hormone (ADH) release, such as the syndrome of inappropriate ADH secretion (SIADH). The use of intravenous fluids in these patients should prompt caution, as the inability of the kidneys to properly dilute the urine can easily result in deterioration of hyponatremia.

**Methods:**

In this report, a transparent and clinically applicable equation is derived that can be used to calculate the estimated effect of different types and volumes of crystalloid infusate on the serum sodium concentration in SIADH patients. As a “proof of concept”, we discuss five SIADH patient cases from our clinic. Alternatively, our mathematical model can be used to determine the infusate volume that is required to produce a certain desired change in the serum sodium concentration in SIADH patients.

**Conclusion:**

The presented model facilitates rational intravenous fluid therapy in SIADH patients, and provides a valuable addition to existing prediction models.

## Introduction

The syndrome of inappropriate antidiuretic hormone secretion (SIADH) is characterized by aberrant, feedback-independent secretion of antidiuretic hormone (ADH) by the posterior pituitary gland. ADH stimulates the insertion of aquaporin-2 channels in the apical membrane of collecting duct epithelial cells, which results in the renal retention of pure water [1]. Because of the tonic ADH secretion in SIADH, it is characterized by a relatively fixed level of urine concentration, which is reflected by a relatively fixed urine osmolarity, and often by hypotonic hyponatremia [2, 3]. The improvident administration of intravenous fluids in SIADH patients frequently exacerbates hyponatremia. As SIADH is a common finding in hospitalized patients, a quantitative insight into the effects of administering intravenous fluids in this disorder is essential for every clinician.

Over the years, a wide range of interesting mathematical models has been derived to predict the effect of intravenous fluid therapy on the serum sodium concentration (most notably the Adrogué–Madias equation), but unfortunately, the vast majority of these models cannot be applied to a patient with a disorder of abnormal renal water-handling [4]. Another model, proposed by Nguyen and Kurtz [5], could theoretically be used to calculate the required amount of intravenous fluid volume in patients with SIADH, but its daunting mathematical complexity discourages its application in the clinical practice. In this report, a novel and comprehensible—and, therefore, clinically more appealing—model is proposed, that provides a quantitative insight on the effects of fluid replacement therapy on the serum sodium concentration in patients with SIADH.

A stepwise derivation is presented below.

## Mathematical derivation

The serum sodium concentration ($\left[ {{\text{Na}}^{ + } } \right]_{\text{s}}$) can be accurately described by the simplified Edelman equation as a function of the total body exchangeable sodium and potassium (${\text{Na}}_{\text{e}}^{ + } + {\text{K}}_{\text{e}}^{ + }$) and the total body water (${\text{TBW}}$): [6, 7]

1 $$\left[ {{\text{Na}}^{ + } } \right]_{\text{s}} = \frac{{{\text{Na}}_{\text{e}}^{ + } + {\text{K}}_{\text{e}}^{ + } }}{\text{TBW}}$$

A change in serum sodium concentration is determined by the change in electrolyte-free total body water, assuming that the total amount of exchangeable sodium and potassium does not change:

2 $$\Delta \left[ {{\text{Na}}^{ + } } \right]_{\text{s}} = \left[ {{\text{Na}}^{ + } } \right]_{\text{s,2}} - \left[ {{\text{Na}}^{ + } } \right]_{\text{s,1}} = \frac{{{\text{Na}}_{\text{e}}^{ + } + {\text{K}}_{\text{e}}^{ + } }}{{{\text{TBW}} + \Delta {\text{TBW}}}} - \frac{{{\text{Na}}_{\text{e}}^{ + } + {\text{K}}_{\text{e}}^{ + } }}{\text{TBW}}$$

In which $\left[ {{\text{Na}}^{ + } } \right]_{\text{s,1}}$ and $\left[ {{\text{Na}}^{ + } } \right]_{\text{s,2}}$ represent the serum sodium concentrations before and after the change in total body water, respectively. Algebraic rearrangement expression of the above produces:

3 $$\Delta \left[ {{\text{Na}}^{ + } } \right]_{\text{s}} = \frac{{{\text{TBW}}\left( {{\text{Na}}_{\text{e}}^{ + } + {\text{K}}_{\text{e}}^{ + } } \right)}}{{{\text{TBW}}\left( {{\text{TBW}} + \Delta {\text{TBW}}} \right)}} - \frac{{\left( {{\text{TBW}} + \Delta {\text{TBW}}} \right)\left( {{\text{Na}}_{\text{e}}^{ + } + {\text{K}}_{\text{e}}^{ + } } \right)}}{{{\text{TBW}}\left( {{\text{TBW}} + \Delta {\text{TBW}}} \right)}} = - \frac{{\Delta {\text{TBW}}\left( {{\text{Na}}_{\text{e}}^{ + } + {\text{K}}_{\text{e}}^{ + } } \right)}}{{{\text{TBW}}\left( {{\text{TBW}} + \Delta {\text{TBW}}} \right)}}$$

4 $$\Delta \left[ {{\text{Na}}^{ + } } \right]_{\text{s}} = - \frac{{\Delta {\text{TBW}}\left( {{\text{Na}}_{\text{e}}^{ + } + {\text{K}}_{\text{e}}^{ + } } \right)}}{{{\text{TBW}}\left( {{\text{TBW}} + \Delta {\text{TBW}}} \right)}} = - \frac{{\Delta {\text{TBW}}\left[ {{\text{Na}}^{ + } } \right]_{\text{s}} }}{{{\text{TBW}} + \Delta {\text{TBW}}}}$$

Because ${\text{TBW}} \gg \Delta {\text{TBW}}$ holds true, the equation above can be reduced to

5 $$\Delta \left[ {{\text{Na}}^{ + } } \right]_{\text{s}} = - \left[ {{\text{Na}}^{ + } } \right]_{\text{s}} \frac{{\Delta {\text{TBW}}}}{\text{TBW}}$$

When administering an intravenous crystalloid fluid volume, the net change in electrolyte-free total body water can be described as the difference between the electrolyte-free total body water intake (${\text{EFWI}}$) and the electrolyte-free total body clearance (${\text{EFWC}}$): [8]

6 $$\Delta {\text{TBW}} = {\text{EFWI}} - {\text{EFWC}}$$

For the purpose of this model, the insensible body water losses (such as through perspiration and evaporative water loss from the respiratory tract) in the period between serum sodium concentration measurements are considered negligible. However, if such losses are significant and known, they can easily be taken into account by adding a factor $- \Delta {\text{TBW}}_{\text{loss}}$ to the right-hand side of Eq. (6).

As opposed to the traditional concept of solute-free water intake and clearance, the physiologically more accurate electrolyte-free water intake and clearance focus on relative tonicity rather than relative osmolarity, and ignore osmotically inert solutes (such as urea). Electrolyte-free total body water intake and the electrolyte-free total body clearance can be calculated as follows: [8, 9]

7 $${\text{EFWI}} = V_{\text{i}} \left( {1 - \frac{{\left[ {{\text{E}}^{ + } } \right]_{\text{i}} }}{{\left[ {{\text{Na}}^{ + } } \right]_{\text{s}} }}} \right)$$

8 $${\text{EFWC}} = V_{\text{u}} \left( {1 - \frac{{\left[ {{\text{E}}^{ + } } \right]_{\text{u}} }}{{\left[ {{\text{Na}}^{ + } } \right]_{\text{s}} }}} \right)$$

Here, $V_{\text{i}}$, $V_{\text{u}}$, $\left[ {{\text{E}}^{ + } } \right]_{\text{i}}$, and $\left[ {{\text{E}}^{ + } } \right]_{\text{u}}$ represent the infusate volume, the urine volume, the cation concentration of the administered crystalloid infusate, and the cation concentration of urine, respectively (in which: $\left[ {{\text{E}}^{ + } } \right]_{\text{i}} = \left[ {{\text{Na}}^{ + } + {\text{K}}^{ + } } \right]_{\text{i}}$ and $\left[ {{\text{E}}^{ + } } \right]_{\text{u}} = \left[ {{\text{Na}}^{ + } + {\text{K}}^{ + } } \right]_{\text{u}}$).

Musch et al. [10] have extensively investigated which urinary parameter best describes renal electrolyte-free water-handling in SIADH patients and most accurately predicts their serum sodium response to saline infusion. It was concluded that the theoretical maximum value for the urine cation concentration ($\left[ {{\text{E}}^{ + } } \right]_{\text{u, max}} = \left[ {{\text{Na}}^{ + } + {\text{K}}^{ + } } \right]_{\text{u,max}}$, which was defined by the authors as the theoretical steady-state of the urine cation concentration after several hours of saline infusion), and not the initial urine cation concentration ($\left[ {{\text{E}}^{ + } } \right]_{\text{u}}$), has the best predictive value for this response (*r* = − 0.81, *p* < 0.001 versus *r* = − 0.51, *p* < 0.05) [10]. This implies that the theoretical maximum urine tonicity ($T_{\text{u,max}}$) most accurately predicts the change in serum sodium concentration due to saline infusion in SIADH patients.

Therefore, Eq. (8) has to be modified as follows:

9 $${\text{EFWC}} = V_{\text{u}} \left( {1 - \frac{{\left[ {{\text{E}}^{ + } } \right]_{\text{u,max}} }}{{\left[ {{\text{Na}}^{ + } } \right]_{\text{s}} }}} \right)$$

Assuming that renal salt-handling is intact in SIADH, the kidneys will excrete the introduced electrolytes [the factor 2 to account for urine anions cancels out on both sides of Eq. (10)]: [11]

10 $$V_{\text{i}} \left[ {{\text{E}}^{ + } } \right]_{\text{i}} = V_{\text{u}} \left[ {{\text{E}}^{ + } } \right]_{\text{u,max}}$$

11 $$V_{\text{u}} = \frac{{V_{\text{i}} \left[ {{\text{E}}^{ + } } \right]_{\text{i}} }}{{\left[ {{\text{E}}^{ + } } \right]_{\text{u,max}} }}$$

Combining the Eqs. (6), (7), (8), (9) and (10) produces

12 $$\Delta {\text{TBW}} = V_{\text{i}} \left( {1 - \frac{{\left[ {{\text{E}}^{ + } } \right]_{\text{i}} }}{{\left[ {{\text{Na}}^{ + } } \right]_{\text{s}} }}} \right) - \frac{{V_{\text{i}} \left[ {{\text{E}}^{ + } } \right]_{\text{i}} }}{{\left[ {{\text{E}}^{ + } } \right]_{\text{u,max}} }}\left( {1 - \frac{{\left[ {{\text{E}}^{ + } } \right]_{\text{u,max}} }}{{\left[ {{\text{Na}}^{ + } } \right]_{\text{s}} }}} \right)$$

13 $$\Delta TBW = V_{i} - V_{i} \frac{{\left[ {E^{ + } } \right]_{i} }}{{\left[ {Na^{ + } } \right]_{s} }} - V_{i} \frac{{\left[ {E^{ + } } \right]_{i} }}{{\left[ {E^{ + } } \right]_{u,\text{max}} }} + V_{i} \frac{{\left[ {E^{ + } } \right]_{i} }}{{\left[ {Na^{ + } } \right]_{s} }} = V_{i} \left( {1 - \frac{{\left[ {E^{ + } } \right]_{i} }}{{\left[ {E^{ + } } \right]_{u,\text{max}} }}} \right)$$

Combining this result with Eq. (5) results in

14 $$\Delta \left[ {{\text{Na}}^{ + } } \right]_{\text{s}} = - \frac{{\left[ {{\text{Na}}^{ + } } \right]_{\text{s}} V_{\text{i}} }}{\text{TBW}}\left( {1 - \frac{{\left[ {{\text{E}}^{ + } } \right]_{\text{i}} }}{{\left[ {{\text{E}}^{ + } } \right]_{\text{u,max}} }}} \right) = \frac{{\left[ {{\text{Na}}^{ + } } \right]_{\text{s}} V_{\text{i}} }}{\text{TBW}}\left( {\frac{{\left[ {{\text{E}}^{ + } } \right]_{\text{i}} }}{{\left[ {{\text{E}}^{ + } } \right]_{\text{u,max}} }} - 1} \right)$$

Infusate tonicity ($T_{\text{i}}$) and urine tonicity ($T_{\text{u}}$) are determined by the osmotically active cations and anions in the infusate and urine, respectively. Therefore, in terms of tonicity, Eq. (14) can be rewritten as follows:

15 $$\Delta \left[ {{\text{Na}}^{ + } } \right]_{\text{s}} = \frac{{\left[ {{\text{Na}}^{ + } } \right]_{\text{s}} V_{\text{i}} }}{\text{TBW}}\left( {\frac{{2\left[ {{\text{E}}^{ + } } \right]_{\text{i}} }}{{2\left[ {{\text{E}}^{ + } } \right]_{\text{u,max}} }} - 1} \right) = \frac{{\left[ {{\text{Na}}^{ + } } \right]_{\text{s}} V_{\text{i}} }}{\text{TBW}}\left( {\frac{{T_{\text{i}} }}{{T_{\text{u,max}} }} - 1} \right)$$

The tonicity of a crystalloid intravenous fluid (which only consists of equal concentrations of cations and anions) is constant and equals twice its cation concentration. It is, therefore, equal to its osmolarity ($O_{\text{i}}$):

16 $$T_{\text{i}} = \left[ {{\text{E}}^{ + } } \right]_{\text{i}} + \left[ {{\text{E}}^{ - } } \right]_{\text{i}} = 2\left[ {{\text{E}}^{ + } } \right]_{\text{i}} = O_{\text{i}}$$

Whereas urine osmolarity remains relatively fixed in SIADH, urine tonicity will change during the infusion of saline due to the renal excretion of the infused electrolytes until a steady-state tonicity $T_{\text{u,max}}$ is reached, which cannot be measured prior to infusate administration. However, $T_{\text{u,max}}$ can be fairly reliably estimated as a percentage of the initial urine osmolarity (i.e., before infusate administration) [9, 10]. Both Shimizu et al. [9] and Musch et al. [10] have experimentally established that—for any given urine osmolarity—the $T_{\text{u,max}}$ of SIADH patients constitutes approximately 60% of the initial urine osmolarity under normal dietary conditions; they concluded that $2\left[ {{\text{Na}}^{ + } + {\text{K}}^{ + } } \right]_{\text{u,max}} /O_{\text{u}}$ equals 59.7 ± 1.7%, and that $\left[ {{\text{Na}}^{ + } + {\text{K}}^{ + } } \right]_{\text{u,max}} /O_{\text{u}}$ equals 33.0 ± 10.0%, respectively. The remaining 40% consists of osmotically inert solutes, such as urea [9, 10]. Therefore,

17 $$T_{\text{u,max}} = \left[ {{\text{E}}^{ + } } \right]_{\text{u, max}} + \left[ {{\text{E}}^{ - } } \right]_{\text{u, max}} = 2\left[ {{\text{E}}^{ + } } \right]_{\text{u, max}} \approx 0.6O_{\text{u}}$$

Because the urine osmolarity in SIADH is feedback-independent and relatively fixed for a given patient, so is the corresponding maximum urine tonicity [2, 3]. The expression above is in line with the clinical observations by—among others—Hoorn et al. [12], Zietse et al. [13] and Shimizu et al. [9] that isotonic saline can be an effective treatment for SIADH if, and only if, the initial urine osmolarity is lower than 530 mOsmol/L. The osmolarity of normal saline (308 mOsmol/L) equals approximately 60% of 530 mOsmol/L. In other words, saline infusion will raise the serum sodium concentration in SIADH as long as its tonicity is higher than the maximum urine tonicity for a given urine osmolarity.

Substitution of the results from Eqs. (16) and (17) in Eq. (15) produces the following relationship:

18 $$\Delta \left[ {{\text{Na}}^{ + } } \right]_{\text{s}} = \frac{{\left[ {{\text{Na}}^{ + } } \right]_{\text{s}} V_{\text{i}} }}{\text{TBW}}\left( {\frac{{T_{\text{i}} }}{{T_{\text{u,max}} }} - 1} \right) = \frac{{\left[ {{\text{Na}}^{ + } } \right]_{\text{s}} V_{\text{i}} }}{\text{TBW}}\left( {\frac{{O_{\text{i}} }}{{0.6O_{\text{u}} }} - 1} \right)$$

19 $$\Delta \left[ {{\text{Na}}^{ + } } \right]_{\text{s}} = \frac{{\left[ {{\text{Na}}^{ + } } \right]_{\text{s}} V_{\text{i}} }}{\text{TBW}}\left( {1.7\frac{{O_{\text{i}} }}{{O_{\text{u}} }} - 1} \right)$$

In line with the Adrogué–Madias equation, Eq. (19) can be further simplified for an infusate volume of 1 L (i.e., $V_{\text{i}} = 1$):

20 $$\Delta \left[ {{\text{Na}}^{ + } } \right]_{\text{s}} = \frac{{\left[ {{\text{Na}}^{ + } } \right]_{\text{s}} }}{\text{TBW}}\left( {1.7\frac{{O_{\text{i}} }}{{O_{\text{u}} }} - 1} \right)$$

Alternatively, Eq. (19) can easily be rewritten algebraically to determine the infusate volume that is required to cause a certain desired change in the serum sodium concentration ($\Delta \left[ {{\text{Na}}^{ + } } \right]_{\text{s,d}}$) in SIADH patients:

21 $$V_{\text{i}} = \frac{{\Delta \left[ {{\text{Na}}^{ + } } \right]_{\text{s,d}} O_{\text{u}} {\text{TBW}}}}{{\left[ {{\text{Na}}^{ + } } \right]_{\text{s}} \left( {1.7O_{\text{i}} - O_{\text{u}} } \right)}}$$

## Discussion and conclusion

In the previous section, a novel and straightforward equation has been derived that can be useful to estimate the effect of intravenous fluid therapy on the serum sodium concentration in SIADH patients. As mentioned, the use of intravenous fluids in this patient category should prompt caution, as the inability of the kidneys to properly dilute the urine can easily result in deterioration of hyponatremia [2, 3]. Previously described mathematical prediction models, such as the well-known Adrogué–Madias equation, only look at input, whereas output is neglected [14–16]. Therefore, they cannot be applied to patients with abnormal renal water-handling [14–16]. Owing to its mathematical transparency, the presented equation provides ‘bedside’ guidance on fluid replacement therapy in patients with disorders of autonomous vasopressin secretion.

As a “proof of concept”, we have collected five patient examples from our clinic (Table 1). In all of these patients the diagnosis of SIADH was made, based on elevated urinary sodium excretion (> 30 mmol/L), and elevated urine osmolarity—indicating (inappropriate) ADH-mediated free water retention. These patients did not use diuretics, and both hypothyroidism and adrenal insufficiency (or other forms of renal salt-wasting) were ruled out on clinical and biochemical grounds, as these conditions would have perturbed the diagnosis of SIADH. One of these case examples will be discussed in more detail below to demonstrate how the calculation of the expected change in serum sodium concentration is performed.

**Table 1:** Serum sodium concentration response to infusate in five SIADH atients

Patient	A	B	C	D	E
Sex, age	M, 59 years old	M, 79 years old	F, 82 years old	M, 59 years old	F, 85 years old
$\left[ {{\text{Na}}^{ + } } \right]_{\text{s,1}}$	129 mM	133 mM	128 mM	106 mM	122 mM
$\left[ {{\text{Na}}^{ + } } \right]_{\text{s,2}}$	128 mM	131 mM	128 mM	107 mM	124 mM
$O_{\text{s}}$	269 mOsM	271 mOsM	263 mOsM	216 mOsM	264 mOsM
$\Delta \left[ {{\text{Na}}^{ + } } \right]_{\text{s,m}}$	− 1 mM	− 2 mM	0 mM	+ 1 mM	+ 2 mM
$\Delta \left[ {{\text{Na}}^{ + } } \right]_{\text{s,p}}$	− 1.15 mM	− 1.97 mM	+ 0.40 mM	+ 0.88 mM	+ 2.10 mM
$O_{\text{u}}$	890 mOsM	766 mOsM	496 mOsM	336 mOsM	345 mOsM
$\left[ {{\text{Na}}^{ + } } \right]_{\text{u}}$	77 mM	143 mM	127 mM	33 mM	90 mM
Infusate	0.9%-NaCl	0.9%-NaCl	0.9%-NaCl	2.5%-NaCl	2.5%-NaCl
$O_{\text{i}}$	308 mOsM	308 mOsM	308 mOsM	856 mOsM	856 mOsM
$V_{\text{i}}$	1.0 L	1.5 L	1.5 L	0.15 L	0.15 L
${\text{TBW}}$	46L	32L	27L	60L	28L
Primary diagnosis	Incisional hernia	Mediastinitis	Sjögren’s syndrome	Lingering pneumonia	Viral RTI
Secondary diagnosis	SIADH, drug-induced	SIADH, drug-induced	SIADH	SIADH	SIADH, drug-induced

*Patient A* is a 59-year-old male with a documented case of bipolar disorder, who was admitted to the surgery ward because of an incisional hernia. The patient has a body weight of 77 kg, which corresponds to an estimated total body water of 46 L. The surgeon consults the internist because the serum sodium concentration of this patient has dropped after the administration of normal saline. Upon admittance, his serum sodium concentration is 129 mmol/L and his serum osmolarity is 269 mOsmol/L. His urine osmolarity on admission is 890 mOsmol/L with a urine sodium concentration of 77 mmol/L. The patient does not use diuretics and both hypothyroidism and hypocortisolism are ruled out on clinical and biochemical grounds. Therefore, the diagnosis of hypotonic hyponatremia due to SIADH is made, most likely as a result of his long-term use of quetiapine. At the moment of consultation, the patient had already received 1 L of normal saline. The effect of administering 1 L of normal saline (with an uncorrected osmolarity of approximately 308 mOsmol/L) on his serum sodium concentration can easily be predicted by inserting the above-mentioned values in Eq. (19):

22 $$\Delta \left[ {{\text{Na}}^{ + } } \right]_{\text{s}} = \frac{{\left[ {{\text{Na}}^{ + } } \right]_{\text{s}} V_{\text{i}} }}{\text{TBW}}\left( {1.7\frac{{O_{\text{i}} }}{{O_{\text{u}} }} - 1} \right) = \frac{129 \cdot 1.0}{46}\left( {\frac{1.7 \cdot 308}{890} - 1} \right) = - 1.15$$

This calculation shows that the expected change in his serum sodium concentration, according to our mathematical model, is − 1.15 mmol/L, which means that administering normal saline should exacerbate his pre-existing condition.

On the other hand, according to the Adrogué–Madias equation the expected change in serum sodium concentration would be [4]

23 $$\Delta \left[ {{\text{Na}}^{ + } } \right]_{\text{s}} = \frac{{\left[ {{\text{Na}}^{ + } + {\text{K}}^{ + } } \right]_{\text{i}} - \left[ {{\text{Na}}^{ + } } \right]_{\text{s}} }}{{{\text{TBW}} + 1}} = \frac{154 - 129}{46 + 1} = 0.53$$

which means that the Adrogué–Madias equation predicts that the serum sodium concentration will increase with 0.53 mmol/L, rather than decrease.

The evening after administration of the normal saline, blood is drawn again. His new serum sodium concentration turns out to be 128 mmol/L. This measured change in serum sodium concentration of − 1 mmol/L corresponds to the change that was predicted by our equation. Because the Adrogue–Madias equation only focuses on the administered infusate and does not take renal water- and salt-handling into account, it will incorrectly predict the change in serum sodium concentration in disorders characterized by tonic ADH secretion, such as SIADH.

As can be seen in Table 1, the presented equations accurately predict the measured change in serum sodium concentration in these five SIADH patients for different types and different volumes of saline infusion. A second measurement of serum sodium concentration was performed several hours after the intravenous fluid volume had been completely administered to allow renal handling of the infusate. The included patient cases have been selected retrospectively from various wards, as we consider it unethical to deliberately administer a type of infusate that would likely exacerbate their conditions according to our model.

The derived mathematical model primarily rests on the notion that the change in electrolyte-free total body water—and, therefore, the change in serum sodium concentration—results from the imbalance between the electrolyte-free total body water intake and the electrolyte-free total body clearance [2, 3, 8, 9]. The ratio of infusate tonicity to maximum urine tonicity defines whether a certain infusate volume represents a net electrolyte-free body water load or a net electrolyte-free body water loss, which intuitively stands to reason. Indeed, in the previously discussed case of normal saline infusion in a patient with SIADH—who produces very concentrated urine—it can easily be seen that $T_{\text{i}} /T_{\text{u,max}} < 1$, which means that this type of infusate will aggravate the pre-existing hypotonic hyponatremia (as was the case in the aforementioned example). Even in SIADH—which is classically characterized by tonic ADH secretion—the secretion of ADH will most likely fluctuate to some extent and the urine osmolarity will not remain entirely constant. Therefore, the urine osmolarity measured in urine collected over a longer time period (e.g., 24-h urine collection) will most likely yield more reliable values than the urine osmolarity measured in a spot urine sample. However, 24-h urine collection is labor-intensive and not always feasible if rapid ‘bedside’ decisions regarding intravenous fluid therapy are required. Furthermore, it can reasonably be assumed that the renal handling of electrolyte-free water in SIADH will not fluctuate to a clinically significant degree during the relatively short period of time between serum sodium measurements, in which the kidneys process the administered infusate [2, 3]. Therefore, if the time between the measurement of urinary indices and the administration of an infusate is relatively short, we recommend measurement of the urine osmolarity in a spot urine sample.

As mentioned before, the presented equation should only be used to calculate infusate-induced changes in serum sodium concentration in disorders characterized by tonic ADH secretion (most notably SIADH, but it could theoretically also be applied to the reset osmostat syndrome, to diabetes insipidus, and to those receiving vasopressin as a part of a treatment for circulatory shock). The proposed model is not suited to be applied to patients with a disorder of aberrant ADH secretion in which hypovolemia is the primary stimulus for ADH release (e.g., intravascular volume depletion due to diuretic use, adrenal insufficiency, extra-renal volume loss, heart failure with forward failure, and cirrhosis), since administering intravenous fluid will correct hypovolemia and remove the ADH secretion stimulus [12]. In this case, the urine osmolarity—and therefore, $T_{\text{u,max}}$—can no longer be assumed to be fixed following infusion. Furthermore, in patients with significant extra-renal water loss (e.g., considerable perspiration) or significant water gain (e.g., psychogenic polydipsia), the total body water balance as described in Eq. (6) will be inaccurate.

In conclusion, the presented model is a useful and transparent clinical tool to predict the effect of fluid replacement therapy in patients with SIADH (and potentially in patients with other disorders of tonic ADH secretion). The equations can be used as a means for clinicians to get a quantitative ‘order-of-magnitude’ understanding of how intravenous crystalloid fluids will influence the serum sodium concentration in these patients, in which both input and output are considered. That being said, validation of our model in a larger patient cohort and in different clinics is desirable.

## List of symbols

ADH: Antidiuretic hormone
SIADH: Syndrome of inappropriate antidiuretic hormone secretion
${\text{Na}}_{\text{e}}^{ + } + {\text{K}}_{\text{e}}^{ + }$: Total body exchangeable sodium and potassium
$\left[ {{\text{Na}}^{ + } } \right]_{\text{s,1}}$: Serum sodium concentration before intravenous fluid
$\left[ {{\text{Na}}^{ + } } \right]_{\text{s,1}}$: Serum sodium concentration after intravenous fluid
$O_{\text{s}}$: Serum osmolarity before intravenous fluid
$\Delta \left[ {{\text{Na}}^{ + } } \right]_{\text{s,m}}$: Measured change in serum sodium concentration
$\Delta \left[ {{\text{Na}}^{ + } } \right]_{\text{s,p}}$: Predicted change in serum sodium concentration
$\Delta \left[ {{\text{Na}}^{ + } } \right]_{\text{s,d}}$: Desired change in serum sodium concentration
$$O_{\text{u}}$$: Urine osmolarity
$$T_{\text{u}}$$: Urine tonicity
$T_{\text{u,max}}$: Theoretical maximum urine tonicity
$$V_{\text{u}}$$: Urine volume
$\left[ {{\text{Na}}^{ + } } \right]_{\text{u}}$: Urinary sodium concentration
$$O_{\text{i}}$$: Infusate osmolarity
$$T_{\text{i}}$$: Infusate tonicity
$$V_{\text{i}}$$: Infusate volume
${\text{EFWI}}$: Electrolyte-free total body water intake
${\text{EFWC}}$: Electrolyte-free total body water clearance
$\left[ {E^{ + } } \right]_{\text{i}}$: Cation concentration of the administered crystalloid infusate
$\left[ {{\text{E}}^{ + } } \right]_{u}$: Cation concentration of urine
${\text{TBW}}$: Total body water (0.6 times body weight for men, 0.5 times body weight for women)
$\Delta {\text{TBW}}$: Change in total body water
